# Efficient Direct Reduction of Graphene Oxide by Silicon Substrate

**DOI:** 10.1038/srep12306

**Published:** 2015-07-21

**Authors:** Su Chan Lee, Surajit Some, Sung Wook Kim, Sun Jun Kim, Jungmok Seo, Jooho Lee, Taeyoon Lee, Jong-Hyun Ahn, Heon-Jin Choi, Seong Chan Jun

**Affiliations:** 1 Nano-Electro Mechanical Device Laboratory, School of Mechanical Engineering, Yonsei University, Seoul 120-749, South Korea; 2 Global E3 Institute and Department of Materials Science and Engineering, Yonsei University, Seoul 120-749, South Korea; 3 Nanobio Device Laboratory, School of Electrical and Electronic Engineering, Yonsei University, Seoul 120-749, South Korea; 4 School of Electrical and Electronic Engineering, Yonsei University, Seoul 120-749, South Korea; 5 Department of Dyestuff Technology, Institute of Chemical Technology, Matunga, Mumbai-400 019, India

## Abstract

Graphene has been studied for various applications due to its excellent properties. Graphene film fabrication from solutions of graphene oxide (GO) have attracted considerable attention because these procedures are suitable for mass production. GO, however, is an insulator, and therefore a reduction process is required to make the GO film conductive. These reduction procedures require chemical reducing agents or high temperature annealing. Herein, we report a novel direct and simple reduction procedure of GO by silicon, which is the most widely used material in the electronics industry. In this study, we also used silicon nanosheets (SiNSs) as reducing agents for GO. The reducing effect of silicon was confirmed by various characterization methods. Furthermore, the silicon wafer was also used as a reducing template to create a reduced GO (rGO) film on a silicon substrate. By this process, a pure rGO film can be formed without the impurities that normally come from chemical reducing agents. This is an easy and environmentally friendly method to prepare large scale graphene films on Si substrates.

Graphene, a monolayer carbon sheet, has superior electrical and mechanical characteristics such as extremely high electron mobility and Young’s Modulus^1^,^2^,^3^. In addition, graphene also has good optical properties including high optical transparency^4^. These remarkable properties make graphene useful for various applications such as field effect transistors (FETs), memory devices and energy storage devices^5^,^6^,^7^. Graphene has been used in various forms in a number of applications, including as large-scale films, nanosheets, and in 3D foam^8^,^9^,^10^. Large scale graphene film is one of the best alternatives to indium tin oxide (ITO), which is most commonly used in transparent conducting films in the display industry^11^. ITO, however, has significant drawbacks such as high cost, limited resources, and lack of flexibility^12^. Graphene film not only has electrical properties superior to that of ITO, but is also flexible and has excellent mechanical properties^13^.

At this point, many researchers have studied graphene film using a variety of fabrication methods. There are two primary methods of making graphene films presently: chemical vapor deposition (CVD) and GO solution-based methods. To make a graphene film using CVD, complicated transfer steps are required for moving graphene from the catalyst substrate to the target substrate because CVD graphene can be grown on restricted substrates such as copper and nickel metal catalysts^14^. This transfer process can cause significant defects; further, PMMA, used as a supporting material during transfer, can leave a residue^15^. CVD processing also requires high temperature. These drawbacks prevent CVD graphene from being commercialized, although it can make large-scale monolayer graphene films.

For GO, many researchers have studied techniques for formation of uniform graphene films from GO solutions such as spin coating, spray coating and using chemical reactions because solution-based methods are suitable for industrial mass production with low cost. GO, however, has a naturally low conductivity, so GO has to be reduced to obtain a conductive film^16^. Generally, additional chemical reducing agents or high temperature annealing are used to make reduced graphene oxide (rGO) films. Pham *et al*. reported spray-coating a GO film with chemical reduction by pre-mixed hydrazine^17^. Becerril *et al*. suggested spin-coated a GO film followed by thermal reduction^18^. Wang *et al*. presented a Meyer rod-coating process with a pre-mixed palladium chloride reducing agent^19^. Ko *et al*. proposed a microliter scale solution method by meniscus-dragging deposition (MDD) with HI acid vapor reduction^20^. Many other techniques such as layer-by-layer (LbL) assembly, and using paper-type films with vacuum filtration have been studied^21^,^22^.

To reduce GO, thermal and chemical reduction have been widely used^8^. In case of thermal reduction, Go was heated at various atmospheres such as vacuum, Ar and H_2_^23^,^24^,^25^,^26^,^27^. Various chemical reduction methods have been studied with diverse type of reducing agents^28^. Hydrazine is most generally used reducing agent due to its high reduction efficiency^29^. Other reducing agents such as hydroquinone^30^, ethylenediamine^31^, ammonia^32^, sodium borohydride^33^ and potassium hydroxide^34^ have been studied. There are many other chemical reducing agents, but many of those are harmful and toxic chemicals^29^. Therefore, environment-friendly chemical reducing agents such as vitamin C^35^, proteins^36^ and bacteria respiration^37^ also have been researched.

Here, we report a new technique to produce large scale graphene films on silicon substrates. In this simple method, reduction of GO and formation of graphene films occur simultaneously on a silicon wafer without any extra reducing agent or high temperature annealing. Moreover, in a comparison with the previous reducing agents, silicon is widely used materials at various applications, so this study method could be easily applied into graphene-silicon composite applications without other additive.

## Results

**Figure 1:**
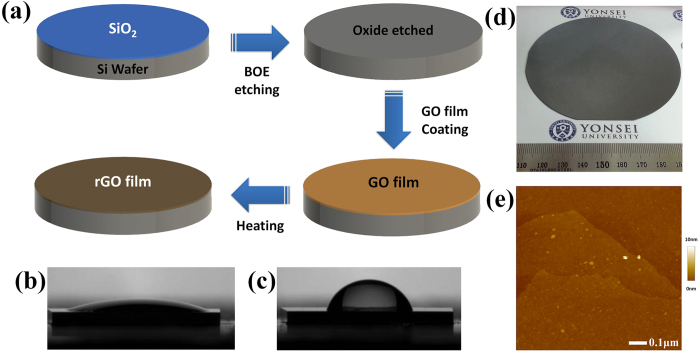
Schematic diagram for experiment steps and film images. (**a**) Schematic flow of direct reduction process by silicon substrate. Oxide layer of silicon wafer was etched by 1:6 buffered oxide etch (BOE). Then, graphene oxide (GO) was coated on bare silicon surface with spray coating method immediately after etching. Finally the wafer was heated up to 100 °C. By this simple process, GO was reduced to graphene oxide (rGO) by silicon. (**b**) The water droplet contact angle on silicon oxide (20.7°), (**c**) on bare silicon (119.7°). (**d**) Photograph of silicon reduced graphene oxide (srGO) film on 4-inch wafer. (**e**) AFM image of srGO film.

Silicon wafers are a commonly used substrate, and graphene is also widely used on silicon substrates. Further, bare silicon can be oxidized easily, so silicon, which acts as a reducing agent in this study, can remove the oxygen groups from GO; as a result, silicon becomes silicon oxide and GO becomes rGO^38^. No further chemicals or treatments like heating are needed to reduce GO by this method. An important advantage of this method is that the resulting rGO has no impurities from chemical reducing agents. Silicon can exist in many forms, including wafers, nanowires or nanosheets^39^,^40^,^41^. In this study, we used nanosheet and wafer-type silicon. The GO film can be reduced on a silicon substrate directly by the process shown in Fig. 1(a). The GO film was coated on surface of oxide etched silicon wafer. The wetting behavior of silicon wafer surface was changed by the oxide layer presence^42^. Before etching process, the surface of silicon wafer was hydrophilic as shown in Fig. 1(b). After fully etching of oxide layer, the surface became hydrophobic as presented in Fig. 1(c). Figure 1(d) shows photographs of uniform silicon reduced graphene oxide (srGO) films made on a 4-inch wafer. Figure 1(e) presents an AFM image of the srGO film. The srGO film has high uniformity with low roughness. The thickness of srGO was 2 nm, and the surface root mean square roughness (Rq) was 0.736 nm.

Silicon nanosheets (SiNSs) prepared by a CVD method^43^ can also be used to reduce GO. SiNSs have a 2-dimentional morphology with a large surface area, and GO also has a similar 2D structure^44^. This similar morphology can increase the contact area between GO and SiNSs, which can enhance the reduction effect. In a first approach, rGO powder was prepared with SiNSs.

**Figure 2:**
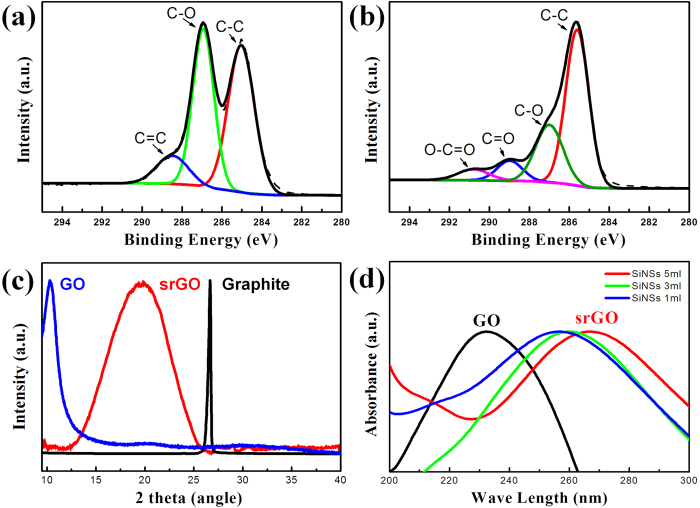
Characterization of silicon reduced graphene oxide (srGO). (**a**) High resolution C1s spectra of GO. (**b**) High resolution C1s spectra of srGO. (**c**) Powder XRD patterns of srGO (red), GO (blue) and graphite (black). (**d**) UV-VIS absorption spectra of srGO (red) and GO (blue).

The reduction effect of silicon to GO was confirmed by X-ray photoelectron spectroscopy (XPS) in Fig. 2(a,b). Chemical bonds between carbon and oxygen were much greater in a GO solution than in the srGO, which proves the successful reduction by silicon. GO has C-C (284.5 eV), C-O (286.7 eV), and C=O (288.3 eV) peaks, and C-O and C=O in srGO were reduced dramatically after reduction. No contaminants (e.g., sodium) were found by XPS analysis in Figure S1. The O1s peak (~530 eV) in srGO is lower than that of GO. The C/O ratio of GO was 2.12 and that of srGO was ~9.32.

Atomic structure change between GO and srGO was determined by X-ray diffraction. Graphite, GO, and srGO powder were measured. Graphite had a sharp peak at 26°, and the peak of GO, after chemical exfoliation from graphite, was shifted to 10°. These peak changes mean the interlayer distance between the carbon layer was increased, i.e., GO was successfully exfoliated^45^. The XRD peak of srGO was at 19.8°, larger than that of GO. This phenomenon corresponds with a reported XRD peak change when GO is reduced^46^. The increase in peak position in GO implies a decrease in the GO interlayer distance. These alterations were due to a decrease in oxygen-containing functional groups, which supported a larger interlayer distance in GO. To confirm the reducing ability of silicon, UV/vis absorbance of GO and srGO was checked. Figure S2 (a) presents optical absorption spectra of pure SiNSs and a GO solution. Before the reduction, GO has an optical peak near 230 nm due to π → π* transitions of aromatic C-C bonds^47^. After the reduction, the optical peak of srGO was shifted to around 270 nm. Figure S3 shows statistical distribution of optical peaks of srGO solutions. The mean value of optical peaks was 267 nm with a small standard derivation (0.7942). The reproducibility of our method was demonstrated statistically. With increasing amounts of SiNSs, the optical peak of srGO was red-shifted. This red shift was caused by electronic conjugation within GO sheets recovered during the reduction process^48^. The wide range graph of optical absorption is shown in Figure S2 (b). This optical property change is in agreement with previous literature^49^. The Raman spectra of GO and srGO powder were analyzed, Figure S4. Commonly, GO has two peaks, a G peak (1341 cm^−1^) and a D peak (1573 cm^−1^). The G peak corresponds to sp^2^ hybridized carbon-carbon bonds, related to first-order scattering of the E_2g_ phonon in graphene and the D peak correlates to lattice distortions, related to a breathing mode of k-point photons of A_1g_ symmetry^46^,^50^. The ratio of these two peaks can be increased due to changes in the degree of reduction, as reported in many previous studies^51^,^52^,^53^. Usually, GO has an ID/IG value of less than 1, and rGO has a value more than 1. This phenomenon is caused by decreased sp2 domains due to reduced size of GO sheets after reduction^8^,^54^,^55^. The incomplete recovery of sp3 defects after reduction reactions also could affect I_D_/I_G_ ratio increase^50^. In this study, the I_D_/I_G_ ratio of GO was 0.80 and that of srGO was 1.11, which is in good agreement with the literature. The ID/IG ratio distribution is shown in Figure S5. By these characterizations, the valuable reducing capability of silicon to GO was confirmed.

**Figure 3:**
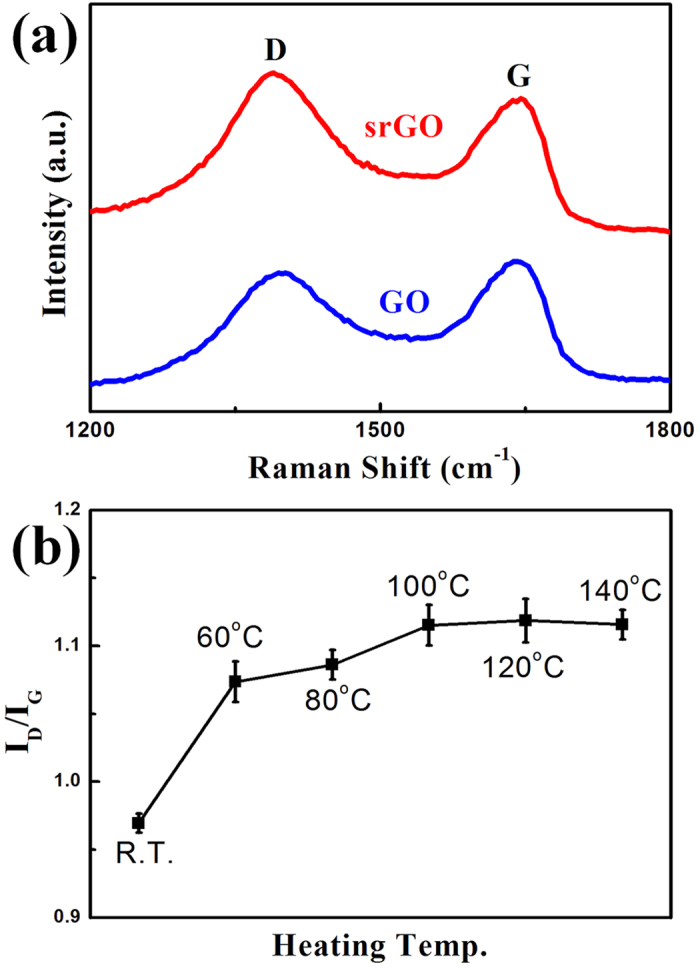
Raman Characteristics of srGO film. (**a**) Raman spectrum of srGO film (red) and GO film (blue). (**b**) ID/IG ratio versus heating temperature from room temperature (rt) to 140 °C. The effect of heating temperature is saturated at 100 °C. Error bars present the standard deviation.

Based on this evidence, a silicon wafer was also used as an efficient template to produce graphene films. The GO film, which was formed on a bare silicon wafer, can be reduced by the substrate alone. The silicon wafer, which has a native oxide layer, was etched this using 1:6 BOE and was rinsed with DI water. After the oxide etching process, the GO solution was spray coated onto the etched wafer surface immediately and was heated. As a result, the as-formed GO film on the silicon substrate was reduced to a srGO film with no further treatment or reducing agent. Film formation and reduction was done simultaneously. The resulting graphene film was investigated using Raman spectroscopy. Figure 3(a) shows a Raman spectrum of the graphene film on the native oxide etched silicon wafer and a non-etched one. As shown, the etched silicon substrate has a reduction effect similar to the SiNSs. The I_D_/I_G_ ratio of the etched wafer was 1.13 and that of the non-etched wafer was 0.79. 2D peak was observed at ~2700 cm^−1^, which is overtone of D peak, and a D + G peak was also observed at ~2973 cm^−1^, which is a combination of the D peak and G peak, as shown in Figure S6^56^. The 2D peak is sensitive for layer number of graphene^57^. Typically GO has weak intensity 2D peak because it has multiple layered C-O bonds in its matrix and usually GO exists as stack of nanosheets instead of mono layer^58^. So, normally 2D and D + G peaks could not provide accurate information regarding GO studies in comparison to single layer graphene studies^59^. Especially in study of reduction method of GO, the enhancement of 2D peak intensity can be observed depending on decreasing of functional groups from GO as shown Figure S6^60^. The srGO has sharp and large 2D peak in comparison to GO. The intensity ratio of I_2D_/I_D+G_ was also increased after reduction process due to graphitic electronic conjugation recovery as shown in table S1^59^.

I_D_/I_G_ ratio changes versus heating temperature were measured from room temperature (rt) to 140 °C. The I_D_/I_G_ ratio increased with an increase in heating temperature up to 100 °C. After 100 °C, the increase in I_D_/I_G_ ratio was saturated, therefore, the optimized heating temperature was 100 °C.

**Figure 4:**
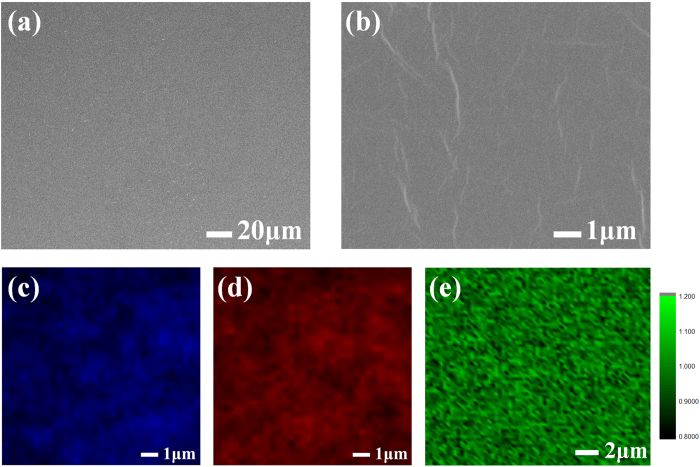
Characteristic SEM and Raman mapping of srGO film. (**a**) Low magnification SEM image of srGO film. (**b**) High magnification SEM image of srGO film. Raman mapping images of (**c**) D-peak and (**d**) G-peak. (**e**) I_D_/I_G_ ratio image.

A graphene film on a 4-inch wafer was formed by our spray coating method. To make a uniform large area GO film, some kind of coating method such as spray coating or spin coating is required to prevent the coffee-ring effect, which occurs at the boundary of the film^61^,^62^. Other coating methods could be applied with the same reduction method presented here if they are suitable for film formation on a silicon wafer. Figure 4(a,b) provide SEM images of the srGO film. A low magnification SEM image displays an overall uniform srGO film coated on the silicon wafer, Fig. 4(a). Local srGO film images are shown in Fig. 4(b) with natural small wrinkles in GO. Figure 4(c,d) shows individual Raman mapping images of d peak and g peak. Figure 4(e) presents uniform I_D_/I_G_ ratio mapping image of srGO.

Sheet resistance was measured to analyze electrical properties of our graphene film. The sheet resistance of the srGO film (3.54 KΩ/square, 2 nm thickness) was considerably lower than that of the GO film (was more than 2 MΩ/square, 5 nm thickness). The srGO sheet resistance is similar to other literature values even though no chemical reducing agent was used. NaOH was also used as an etchant for the silicon wafer to confirm that the reduction effect was not a result of the BOE. All other procedures were kept the same as previously described. Although the etchant was changed, there was no significant change in the Raman spectrum, as shown in Figure S7.

## Discussion

Though the reduction mechanism of GO has been actively studied, but it is not sufficient^29^,^63^. Researchers are mainly using density functional theory (DFT) and molecular dynamics (MD) simulation to investigate the reduction mechanism^64^,^65^,^66^. The reduction reaction includes various chemical reactions in sequence that are not configured in a single reaction^67^. Chemical reactions are different based on the reduction methods^65^,^68^,^69^,^70^. Most current studies have focused to elimination of oxygen containing groups from GO, which are the main purpose of reduction to produce rGO^71^. In this study, we suggest possible mechanism of our method in Figure S8 and S9. GO contains various oxygen functional groups such as hydroxyl, epoxy and ketone groups. These functional groups could be removed from as-made GO by the proposed reduction mechanism as in Figure S9.

Based on the current experiment and characterization results, it is confirmed that GO is reduced by silicon. As the results of our experiments, silicon can absorb oxygen group from GO. That oxygen group could be formed as silicon dioxide. According to XPS data silicon dioxide was formed on silicon wafer after GO reduction as shown in Si 2p spectrums (figure S10)^72^. The SiO_2_ peak (103.65 eV) was found after GO reduction process. As per our hypothesis, we are assuming that the Si-H dangling bonds could be generated on the surface of silicon, which are playing these key roles to reduced oxygen functional groups of GO as shown in Figure S9. Various researchers have been investigated the reduction reaction of oxygenated functional groups by H-terminated silicon surface^73^,^74^,^75^,^76^. The reduction reactions have been conducted by Si-H dangling bonds^77^,^78^,^79^, which were formed during etch process as shown in figure S8^80^,^81^,^82^,^83^. The fluoride ion of remaining very small amount of HF on silicon surface after the etching process, also could help to activate these Si-H bonds^73^. The most of oxygen functional groups could be present as hydroxyl, epoxide and ketone groups as following the Lef-Klinowski model^8^. In addition to this, there are also some carbonyl groups on GO. These active hydrogens on silicon surface reacts with oxygenated functional groups of GO and it causes de-epoxide, de-carbonyl and de-hydroxyl reaction (figure S9) to produce rGO. In case of SiNSs, which have large surface-to-volume ratio, can contain more bonds. It makes SiNSs as good reducing agent. The composite of GO-SiNSs could be used various applications such as Lithium Ion Battery anode. The reaction between silicon wafer and GO film could produce silicon dioxide layer in interlayer of two. Silicon dioxide is one of the best dielectric material, which is important in field of electric device such as field effect transistor. No need to form additional oxide layer to make dielectric layer. It can help our method is applied to various aspects.

This study provides a demonstration of an innovative reduction method for GO. Silicon, a common material widely used throughout industry, can be used to reduce GO. CVD grown 2-dimenional SiNSs can combine with GO sheets due to similar morphology, and this advantage can enhance the reduction effect of silicon. Silicon wafers were also used as reducing templates. By this method, the reduction of GO and film formation occurred simultaneously without any additional chemicals. The graphene film, which is reduced by silicon, has no impurities such as hydrazine or hydrogen iodide, and a high quality graphene film can therefore be achieved. The reproducibility of our method was confirmed statistically. A large scale graphene film can be made by this simple method. The graphene film on a silicon wafer can be transferred to a transparent substrate by a common graphene transfer method. This suggested method is simple, easy, and eco-friendly for graphene film formation and can be used to potentially further commercialization of graphene.

## Methods

### Materials

Silicon wafers were obtained from DASOM RMS (Korea). BOE was purchased from SAMJUN Chemicals (Korea). Graphite was obtained from Bay Carbon (USA). All other chemicals for synthesis GO were obtained from Sigma Aldrich, Korea. All chemicals were used without further purification.

### Experimental procedure

The whole experiment was divided into two categories. The first experiment was conducted as solution process with silicon nanosheets (SiNSs) and GO. After reduction process based on solution, GO was dried as powder form. The second experiment was performed as film process with silicon wafer and GO solutions. The result of this experiment was reduced GO film on silicon wafer.

### Preparation of Graphene Oxide

The graphene oxide solution was prepared by a modified Hummer’s method.

### Preparation of Silicon Nanosheets (SiNSs)

SiNSs were synthesized on Si substrates through chemical vapor deposition using SiCl_4_ as the Si precursor and H_2_ as the carrier gas in high gas flux environment. The substrates were placed at the center of a quartz tube reactor. The reaction temperature was maintained at 1050 °C for 30 min under a H_2_ (99.9999%) and an Ar (99.9999%) atmosphere. Silicon tetra-chloride (SiCl_4_, Aldrich, 99.999%) was introduced into the reactor using a bubbling system. After 30 min, the reactor was cooled to room temperature under an Ar atmosphere.

### Preparation of Reduced Graphene Oxide with SiNSs

SiNS wafers were etched using a 1:6 buffered oxide etch (BOE) for 0.5 seconds to remove the native oxide layer and were dispersed in DI water. A diluted GO solution 25 ml (0.1 mg/ml) and 5 ml of a SiNSs solution (0.1 mg/mg) were mixed well and heated up to 80 °C for 2 hours with mild stirring. Then, the mixed solution was put into a 2 M NaOH solution at 40 °C for 1 day for to fully etch the SiNSs. The solution was washed with DI water by centrifuging 5 times followed by drying.

### Formation of Reduced Graphene Oxide Films

The silicon wafer was put into 1:6 BOE at room temperature for 240 or 320 seconds. Then, the wafer was rinsed with DI water 3 times quickly and dried with flowing N_2_. After drying, the wafer was placed on a spray coating device and heated up to 80 °C. During heating, the GO solution (1 mg/ml) was sprinkled on the wafer surface. The heating was continued up to 2 hours after coating.

### Spray coating of Graphene Oxide

Graphene oxide layers were obtained by facile spray casting onto the functionalized substrate using a double-action airbrush (model GP-70, Sparmax) at a distance of 30 cm with a N_2_ pressure of 29 psi. During the spraying process, the substrate is heated to 100 °C to obtain uniformly deposited graphene oxide layers.

### Characterization

Changes in chemical bonds after reduction were investigated by X-ray photoelectron spectroscopy (XPS, k-alpha, Thermo. U.K.). Adjustments of atomic and molecular structure were probed with X-ray diffraction (XRD, Ultima IV, RIGAKU). The reduction of GO by silicon was analyzed using a Raman Spectrometer (LabRam Aramis, Horriba Jovin Yvon). The absorbance differences between GO and silicon reduced graphene oxide (srGO) and the transmittance of srGO films were measured using a UV/VIS spectrophotometer (V-650, JASCO Corporation). Electrical properties of srGO films were examined by using 4 probe measurement (CMT-SR1000N, AiT). Surface roughness and morphology of the srGO film were investigated using an atomic force microscope (AFM, XE-100, Park Systems) and a field-emission scanning electron microscope (FE-SEM, JEOL-6701F, JEOL Ltd.).

## Additional Information

**How to cite this article**: Chan Lee, S. *et al*. Efficient Direct Reduction of Graphene Oxide by Silicon Substrate. *Sci. Rep*. **5**, 12306; doi: 10.1038/srep12306 (2015).

## Supplementary Material

Supplementary Information
