# Supplementary material for: Efficient Direct Reduction of Graphene Oxide by Silicon Substrate
Source: Sci Rep. 2015 Jul 21;5:12306. doi: 10.1038/srep12306 (PMC4648420; doi:10.1038/srep12306)
Supplement: Supplementary Information [file srep12306-s1.pdf]

SUPPLEMENTARY INFORMATION

Efficient Direct Reduction of Graphene Oxide by Silicon Substrate

Su Chan Lee[a], Surajit Some[a,e]*, Sung Wook Kim[b], Sun Jun Kim[a], Jungmok Seo[c], Jooho Lee[a,f], Taeyoon Lee[c], Jong-Hyun Ahn[d], Heon-Jin Choi[b], Seong Chan Jun[a]*

[a]Nano-Electro Mechanical Device Laboratory, School of Mechanical Engineering, Yonsei University, Seoul 120-749, South Korea

[b]Global E3 Institute and Department of Materials Science and Engineering, Yonsei University, Seoul 120-749, South Korea

[c]Nanobio Device Laboratory, School of Electrical and Electronic Engineering, Yonsei University, Seoul 120-749, South Korea

[d]School of Electrical and Electronic Engineering, Yonsei University, Seoul 120-749, South Korea

[e]Department of Dyestuff Technology, Institute of Chemical Technology, Matunga, Mumbai-400 019, India

[f]Samsung Advanced Institute of Technology, Yongin 446-577, South Korea

Email : scj@yonsei.ac.kr(S.C.J.), someiit2k@gmail.com(S.S.)

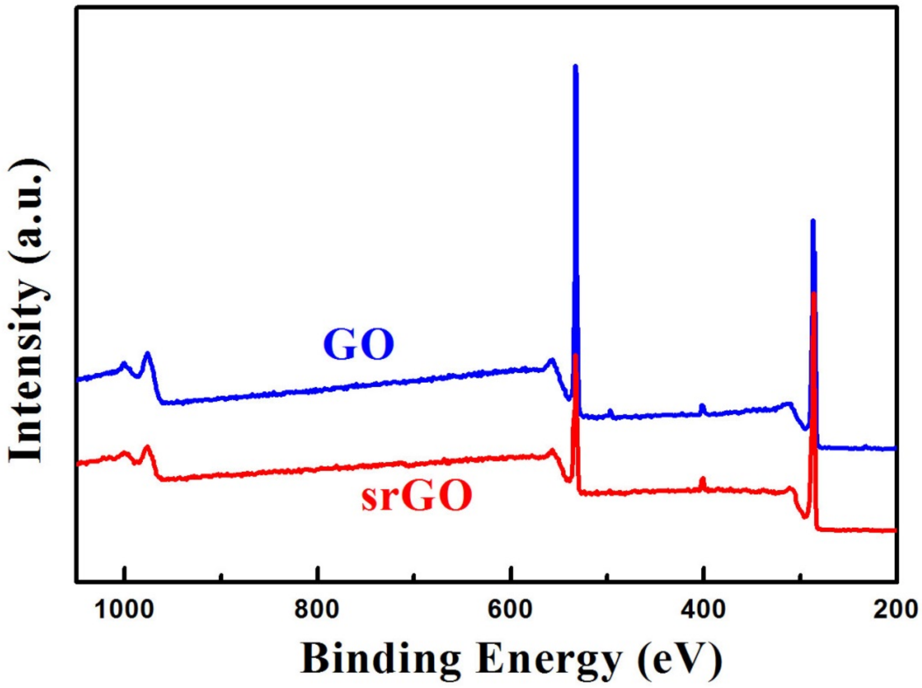

Figure S1. Full range XPS spectra of srGO (red) and GO (blue)

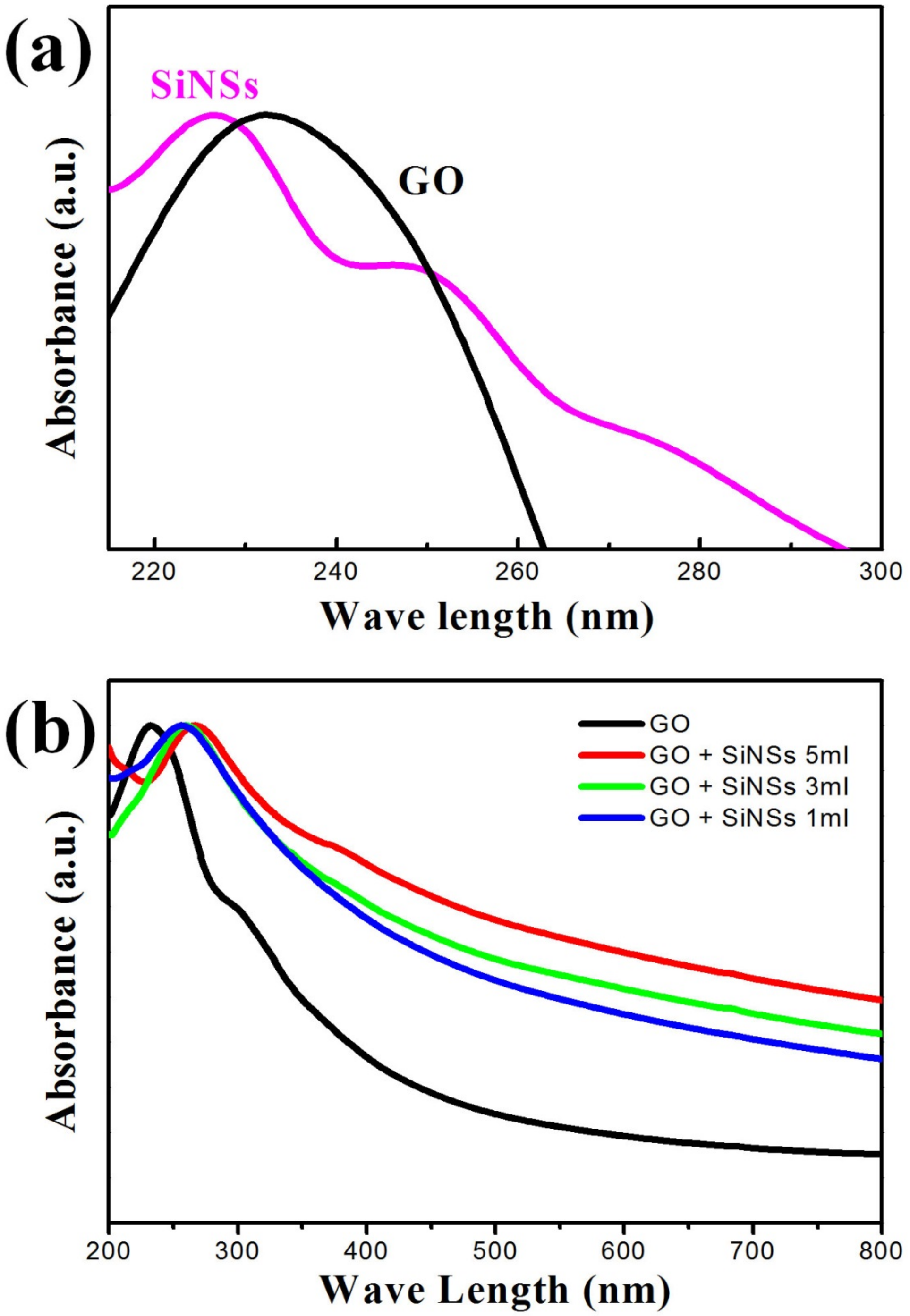

Figure S2. (a) UV/VIS absorption spectra of GO (black) and Silicon NanoSheets (SiNSs, magenta). (b) Wide range UV/VIS absorption spectra of GO and srGO

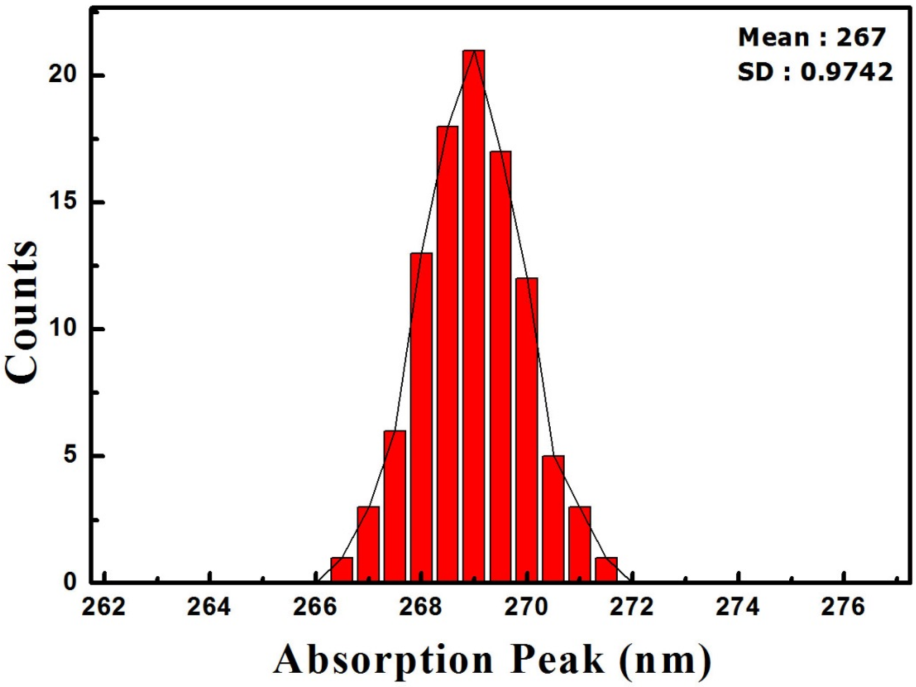

Figure S3. Absorption peak distribution histogram of srGO solution. The mean value is 267nm and standard deviation (SD) is 0.9742.

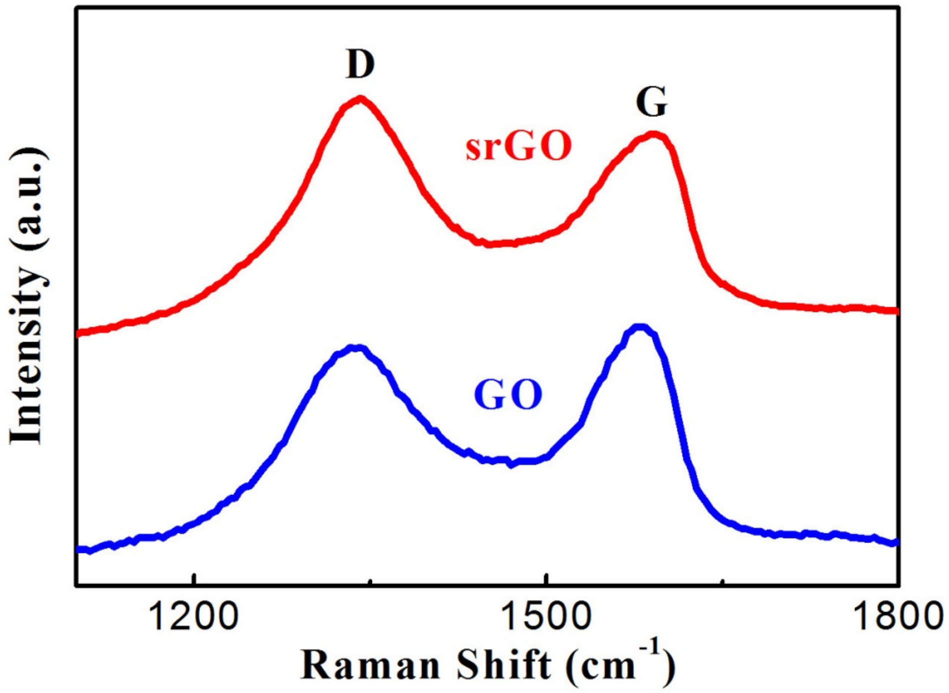

Figure S4. Powder raman spectrum of srGO (red) and GO (blue)

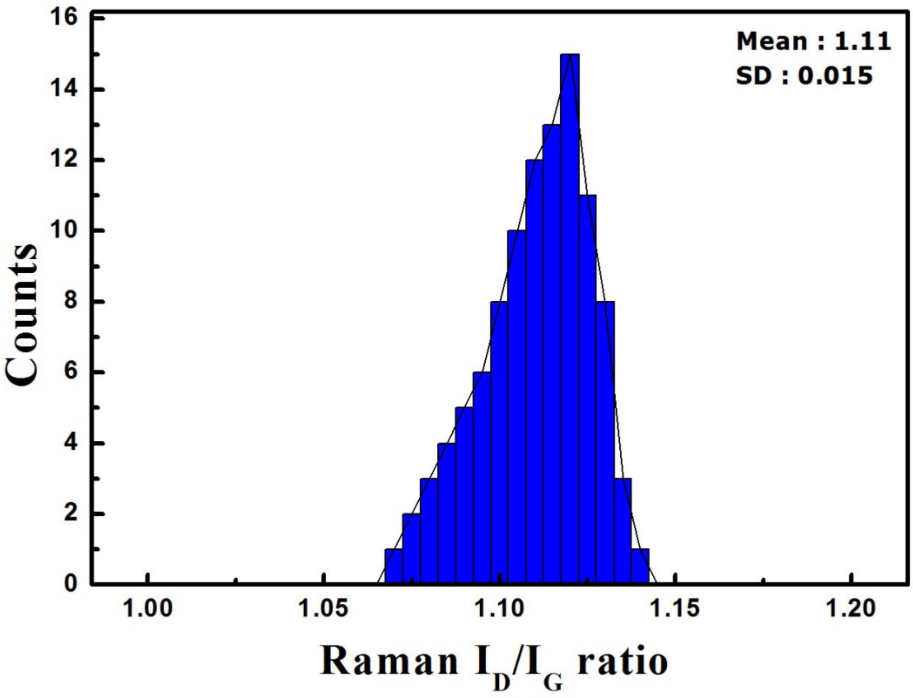

Figure S5. I_D/I_G ratio distribution histogram of srGO powder. The mean value is 1.11 and standard deviation (SD) is 0.015.

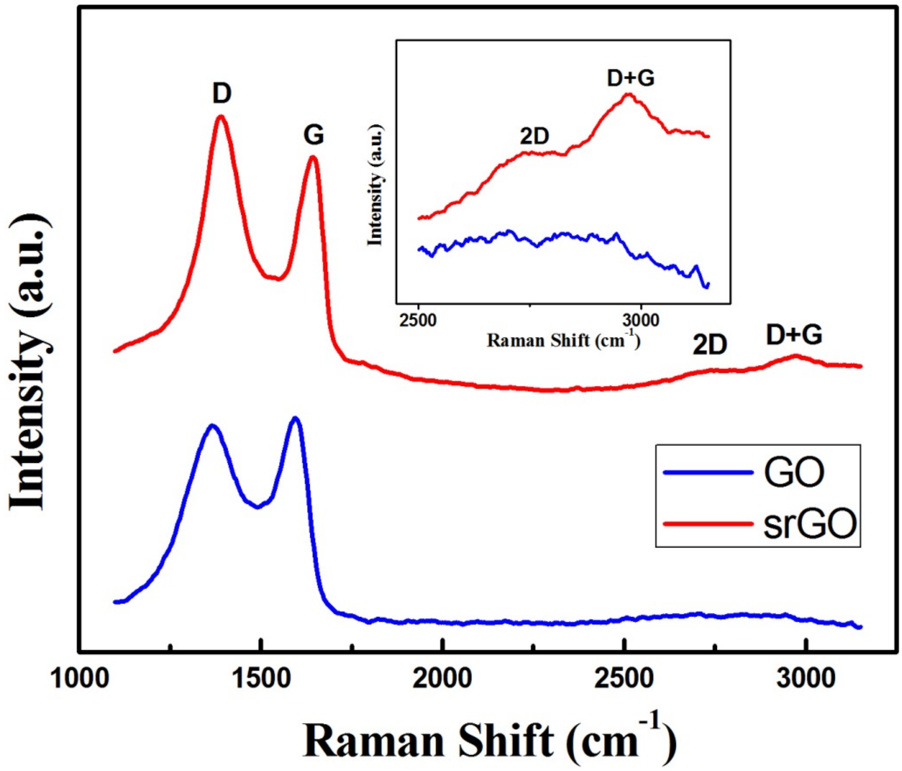

Figure S6. Film raman spectrum of srGO (red) and GO (blue)

	I_D/I_G	I_{2D}/I_{D+G}
Graphene Oxide	0.79	0.98
Reduced Graphene Oxide	1.13	1.69

Table S1. Change of I_D/I_G ratio and I_{2D}/I_{D+G}

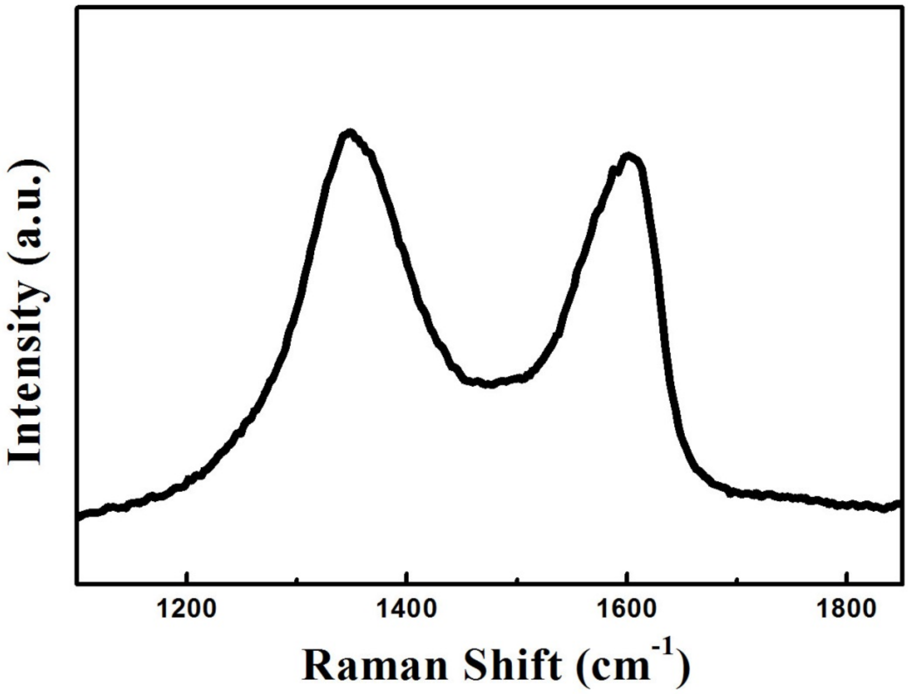

Figure S7. Raman spectrum of srGO (silicon wafer etched by NaOH)

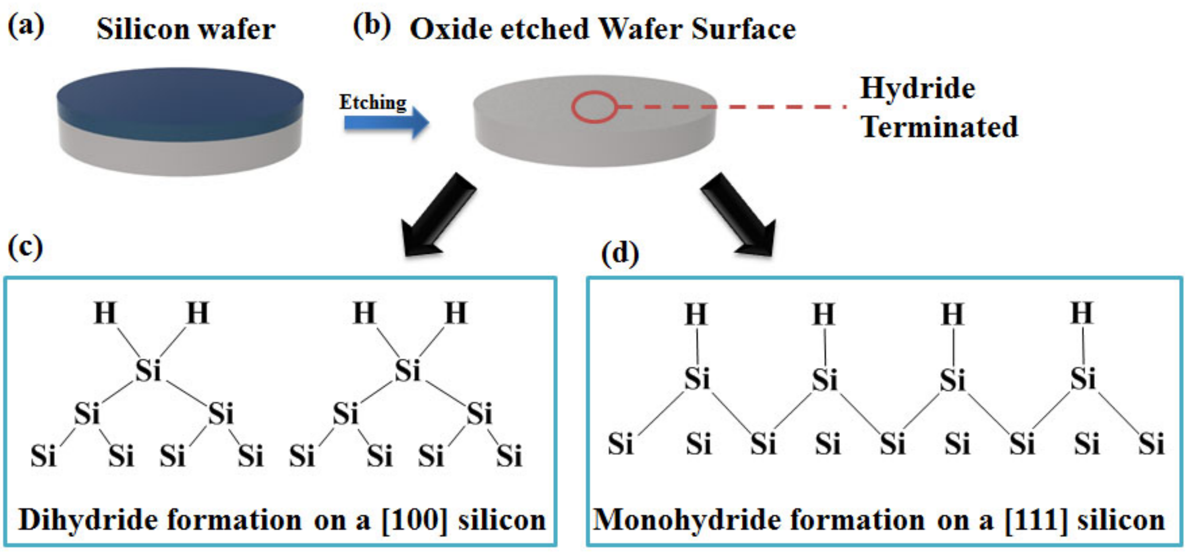

Figure S8. Hydrogen terminated surface formation of silicon wafer

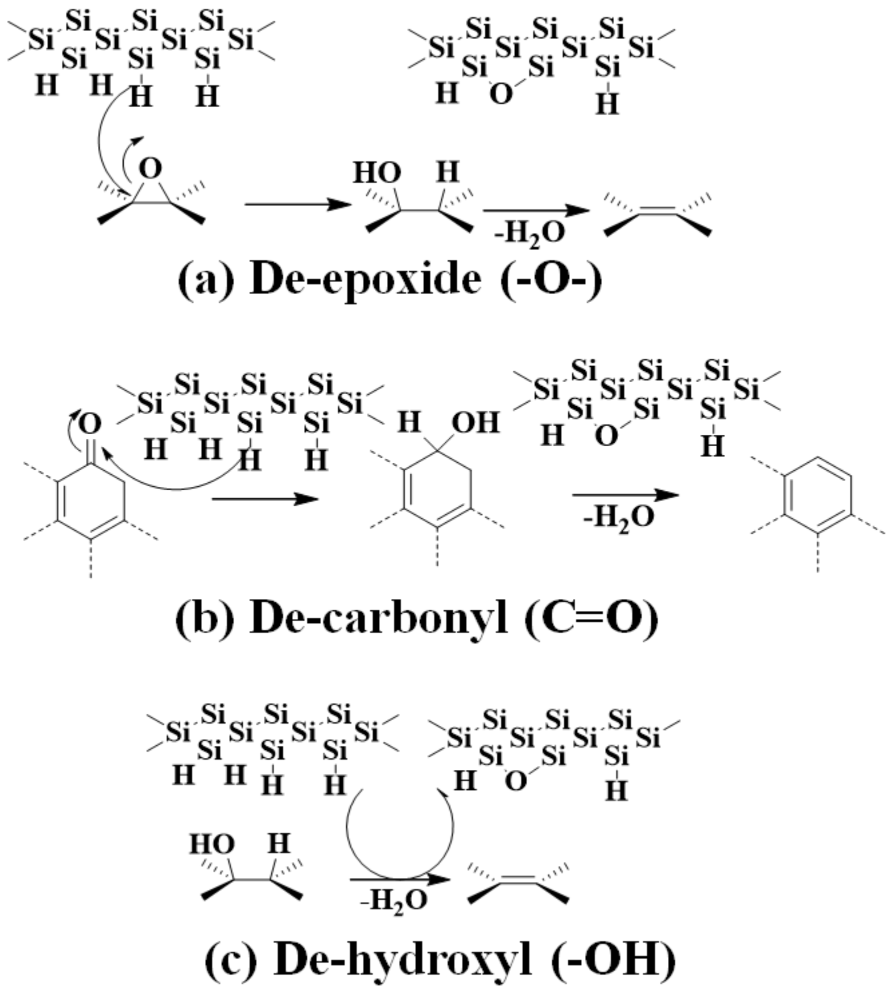

Figure S9. Possible reduction mechanism of graphene oxide with silicon. (a) De-epoxide (b) De-carbonyl (c) De-hydroxyl reaction

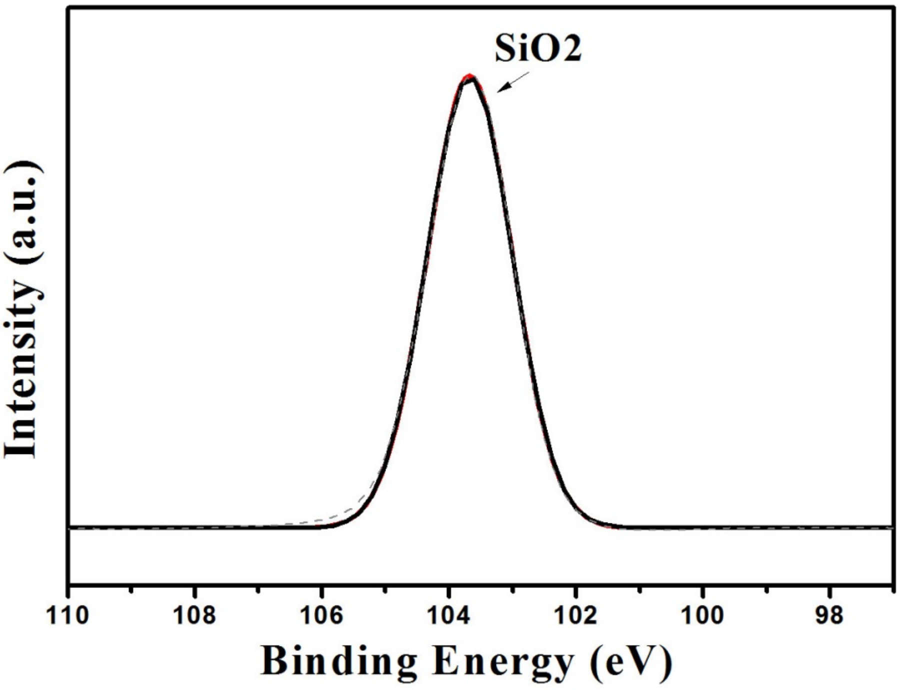

Figure S10. Si2P spectra of srGO.
